# Impact of *Punica granatum* seeds extract (PSE) on renal and testicular tissues toxicity in mice exposed to iron oxide nanoparticles (IONPs)

**DOI:** 10.1038/s41598-024-74410-8

**Published:** 2024-10-30

**Authors:** Yasmin M. Abd El-Aziz, Fatima S. Alaryani, Nesreen Aljahdali, Kamlah Ali Majrashi, Najah M. Albaqami, Marwa S. Khattab, El-Sayed Hemdan Eissa, Zulhisyam Abdul Kari, Ali H. Abu Almaaty

**Affiliations:** 1 Department of Zoology, Faculty of Science, Port Said University, Port Said, 42526 Egypt; 2 College of Science, Department of Biological Sciences, University of Jeddah, 21589 Jeddah, Saudi Arabia; 3 Department of Biological Science, College of Science, King Abdulaziz University, 42742 University Avenues, 21551 Jeddah, Saudi Arabia; 4 Biological Sciences Department, College of Science & Arts, King Abdulaziz University, 21911 Rabigh, Saudi Arabia; 5 Department of Biological Sciences, Faculty of Sciences, King Abdulaziz University, Jeddah, Saudi Arabia; 6 Department of Pathology, Faculty of Veterinary Medicine, Cairo University, Cairo, Egypt; 7 Fish Research Centre, Faculty of Environmental Agricultural Sciences, Arish University, El- Arish, Egypt; 8 Department of Agricultural Sciences, Faculty of Agro-Based Industry, Universiti Malaysia Kelantan, Jeli Campus, 17600 Jeli, Kelantan Malaysia

**Keywords:** Biomarkers, Histochemical, IONPs, Kidney, PSE, Testis, Ecology, Zoology

## Abstract

Recently, nano-manufactured materials have been used to treat many diseases, such as healing wounds and other modern biological applications. This study investigates the positive effect of *Punica granatum* seeds extract on kidney and testicular toxicities induced by iron oxide nanoparticles. Forty mice were randomly divided into four groups; the 1st group was the control group. The 2nd group was dosed daily with PSE at 100 g per kg. The 3rd group was dosed with 10 doses of iron oxide nanoparticles at 30 mg/kg b.wt of a mouse per day, 10 times only, then this toxic substance was withdrawn for the rest of the experimental period (30 days). The 4th group was dosed with the same doses as the second and third groups. In this research, we focused on the possibility of using the positive curative effects of PSE, which were estimated at the level of blood chemistry biomarkers, as well as histological and histochemical examinations for the kidney and testis after exposure of mice to iron oxide nanoparticles. These aim to clarify the effect of iron oxide nanoparticles on kidney and testicular morphology and their functions, as well as the potential ameliorative effects of PSE.

## Introduction

Nanotechnology provides unique ways to raise the absorption and bioavailability of chemicals due to their small size and large surface area^1–3^. The nanoparticles that have been widely used recently, including iron oxide nanoparticles, have become an exciting field of research and study because they are linked to widely published fields such as medical, pharmaceutical, industrial, technological, and other areas due to their unique properties^4,5^. However, there is growing evidence to suggest that exposure to these nanoparticles may lead to toxic and harmful effects^6–8^. Among the possible toxic effects of these nanoparticles, consideration has begun to be given to using *Punica granatum* seed extract (PSE) to reduce kidney and testicular toxicity in mice exposed to iron oxide nanoparticles affecting the organs and tissues of experimental mice^9^.

Toxicity caused by iron oxide nanoparticles in exposed renal and testicular tissues is an area of scientific interest. The kidneys are considered vital organs as they are responsible for removing metabolic wastes by filtering the blood and generating several important hormones^10–13^, while the testicles are essential for producing sperm and regulating male hormones^14,15^. When both organs are exposed to the potential effects of toxic substances in iron oxide nanoparticles, this occurs through the generation of reactive oxygen species (ROS), oxidative stress, and inflammatory cell activity, which can contribute to cell damage and disruption of their vital functions^5,7,16,17^.

According to Gulbins et al.^18^, Baratli^19^, Yarjanli^7^, and Abd El-Aziz et al.^8^ increasing the deposition of iron ion nanoparticles in the cytoplasm of tissues leads to the destruction of mitochondria and increased cell death by enhancing permeability to calcium ions and pre-apoptotic elements such as caspase 9 and cytochrome c, which in turn accelerate the apoptosis process.

In this context, studying the expected positive protective effects of PSE on kidney and testicular toxicity caused by iron oxide nanoparticles in mice is of great importance, as *Punica granatum* seeds are rich in bioactive compounds such as ellagic acid, punicalagin, and anthocyanins, and are characterized by antioxidant, anti-inflammatory, and cell and tissue-protective properties^8,20,21^. These properties make PSE a satisfactory choice for minimizing the harmful effects of iron oxide nanoparticles on experimental vital organs such as the kidneys and testis^22^.

Studies have shown the ability of PSE to reduce oxidative stress, cellular damage, and inflammation caused by certain toxins in various experimental models. Given that the toxicity of iron oxide nanoparticles causes increased oxidative stress and the activity of inflammatory responses, the ability of PSE to counteract these mechanisms could provide a basis for its protective effects against nephrotoxicity and testis exposure to these nanoparticles^8,23,24^.

## Materials and methods

### Ethical approval report

The animal models under the current study were studied based on the approval and reference of the Zoological Ethics Committee at the Faculty of Science, Port Said University, Egypt, with approval report No. ERN: PSU.Sci.31.

### Fe_2_O_3_ nanoparticles (IONPs) preparation

Iron oxide nanoparticles (IONPs) were prepared according to a method published in a previous research review by Abd El-Aziz et al.^8^. A TEM was used to image the iron oxide nanoparticles.

### *Punica granatum* seeds extract (PSE) preparation

Pomegranate fruit was purchased from a market in Port Said, Egypt. The method of Abd El-Aziz et al.^8^ was used to extract the extract by first washing the fruit well and carefully peeling it, separating the seeds, washing and drying them, then grinding them with a grinder (Grinder Retsch, Germany). Then, 50 g of ground seeds were weighed and immersed in 500 ml of methyl alcohol for two days at room temperature, and filtered. This method was repeated two times. Finally, rotary evaporation evaporated the alcohol at 50 °C (Rotavapor, England). After evaporation, the resulting PS extract was divided into Eppendorf tubes and stored in a deep freezer at -5 °C for use in the current study.

### Experimental mice design

**Fig. 1:**
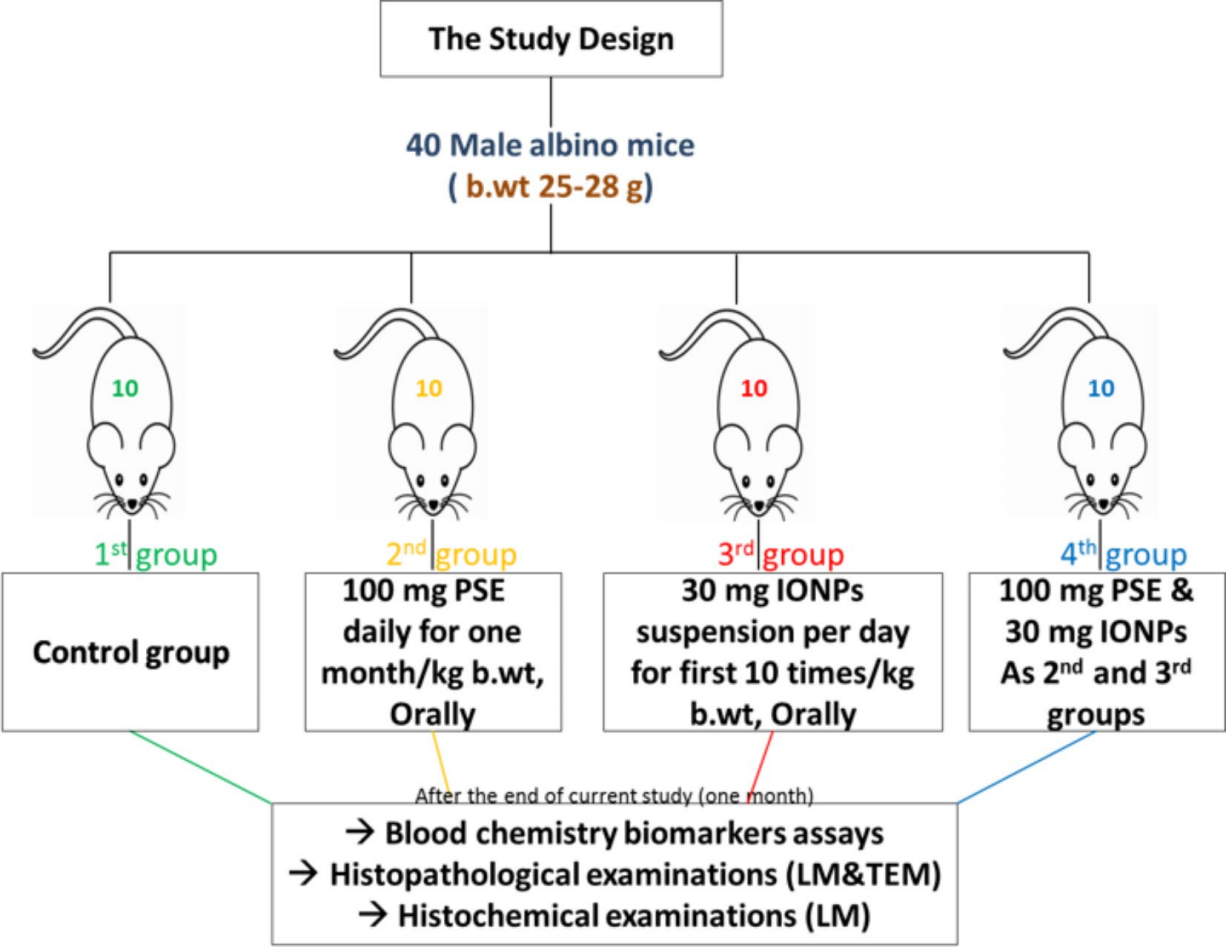
Schematic design showing the current experimental groups and their impact.

40 Male albino mice (*Mus musculus*), weighing 25–28 g, were purchased from the Animal House at the Faculty of Science, Port Said University, Port Said, Egypt. The mice were then raised for a one-week adaptation stage, during which all necessary living conditions were established in plastic boxes, including temperature (25 °C), humidity (52%), a 12:12 h light/dark cycle, diet, water, and good ventilation. After the adaptation period, the experiment began. The mice were randomly distributed and divided into four groups, as shown in Fig. 1.

In the current study, *Punica granatum* seed extract (PSE) was administered at 100 mg per kg of mice daily for a month using oral gavage^25^. Additionally, 30 mg of iron oxide nanoparticles (IONPs) were used per kg of mice, suspended in 100 µl of saline solution (0.99% NaCl), and prepared 10 doses per day, where each mice received 30 mg/kg per day of IONPs dosing during the first 10 days of the current study period. The remaining 20 days of the study period were without doses (withdrawal)^8^. The total duration of the current study (dosing days + withdrawal days) was one month. At the end of the experiment, the mice were fasted for one day, after which they were euthanized by separating the cervical vertebrae with a sharp blade.

Blood was then collected to perform blood chemistry biomarker assays, along with parts of kidney and testicular tissues for tissue sections and histochemistry through optical and electron examinations.

### Blood chemical biomarkers assays

After the mice were euthanized by separating the cervical vertebrae, blood samples were collected from the experimental groups, five from each group (*n* = 5), and placed in tubes containing an anticoagulant agent (EDTA). The samples were then placed in a centrifuge at 4000 rpm for 15 min. The yellow floating layer (serum) was separated and placed in Eppendorf tubes and stored at -20 °C until they were used to measure the blood chemical biomarkers analyzed with a spectrophotometer (Lambda EZ201, Germany). Malondialdehyde (MDA), total bilirubin (TBil), blood urea nitrogen (BUN), creatinine (CREA), and testosterone (TES) were purchased from Biodiagnostic medical company (Giza, Egypt) according to the MDA, TBil, BUN, CREA, and TES kits’ leaflet of the medical company.

### Histopathological examinations

#### Examination of kidney and testicular tissues under the light microscope (LM)

After the euthanized slaughter of the mice, the kidney and testicle tissues were separated and passed through the tissue processing steps. They were placed in a 10% fixative solution (neutral formaldehyde), a series of ethyl alcohol, and xylene, then dipped in paraplast wax to create paraplast wax blocks. These blocks were cut to a thickness of 5 µm using a rotary microtome (*Leica*, Germany), then the tissue ribbons were dyed with H&E dyes and finally examined under a light microscope (LM)^26^.

#### Examination of kidney and testicular tissues under the transmission electron microscope (TEM)

According to Abd El-Aziz et al.^8^ and Abu-Almaaty et al.^27^ method, parts of renal and testicular organs were plucked out for examinations using a transmission electron microscope (JEOL JEM-2100, Japan) at the Electron Microscopy Unit at Mansoura University, Mansoura, Egypt.

### Histochemical examinations

By using Mallory trichrome stain (MTS) as a histochemical stain, we were able to examine the connective tissues, fibers, and amyloid structure after staining kidney and testicular tissues with this specialized dye and examining them under a light microscope^8^.

### Statistical analysis

We can analyze statistics using the SPSS program (20.0) and the One-way ANOVA and Tukey tests to compare the experimental groups with the 1st group as a control group. We will evaluate the means (M) and standard error (SE) to calculate the significance level (*p* < 0.05).

## Results

### TEM for Fe_2_O_3_ nanoparticles (IONPs)

**Fig. 2:**
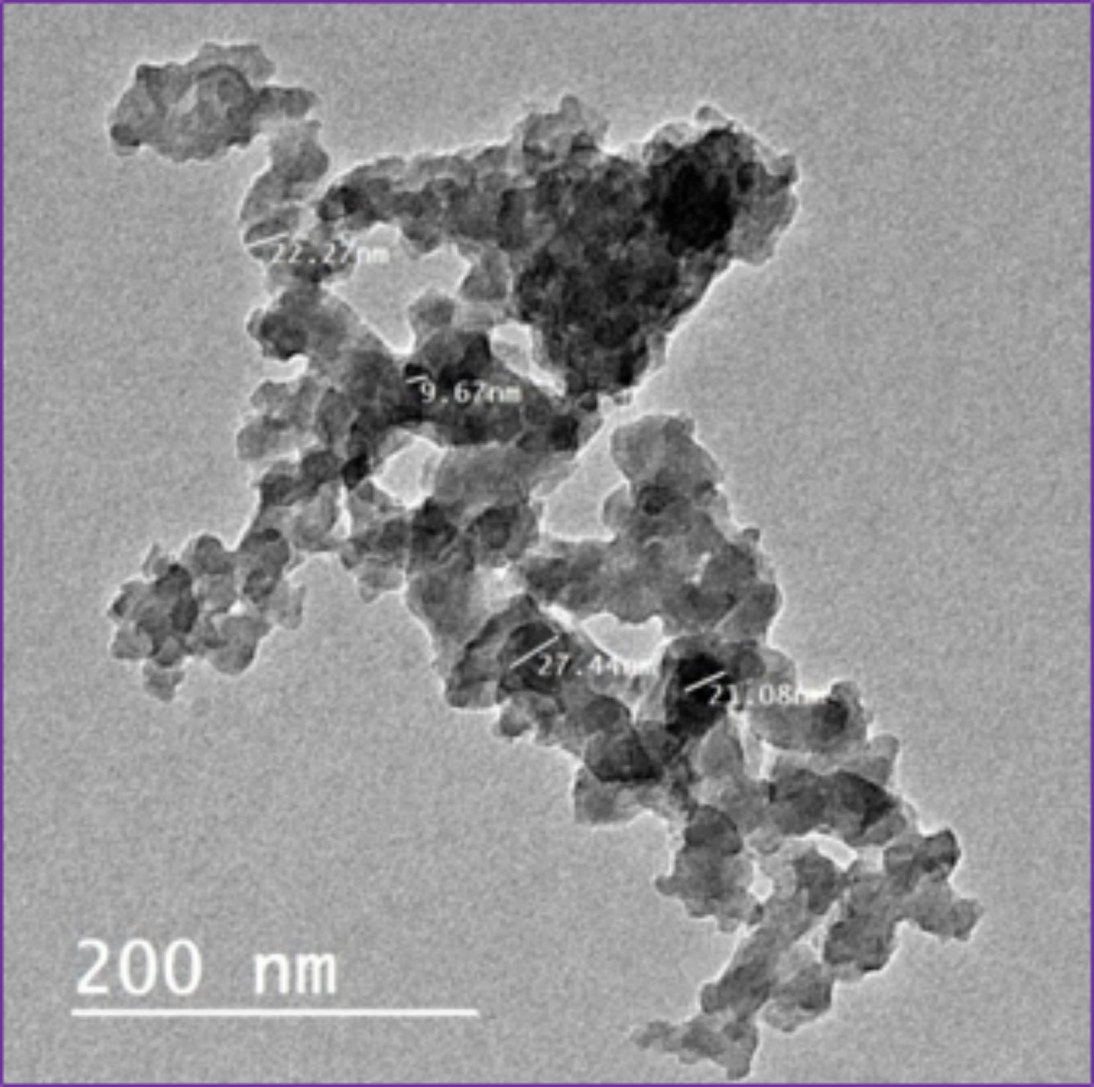
The iron oxide nanoparticles, [TEM, Scale bar = 200 nm].

By using a TEM, spherical iron oxide nanoparticles were demonstrated, and it was found that the range of aggregates ranged from 9.67 to 27.44 nanometers (Fig. 2).

### Blood chemical biomarkers assays

**Fig. 3:**
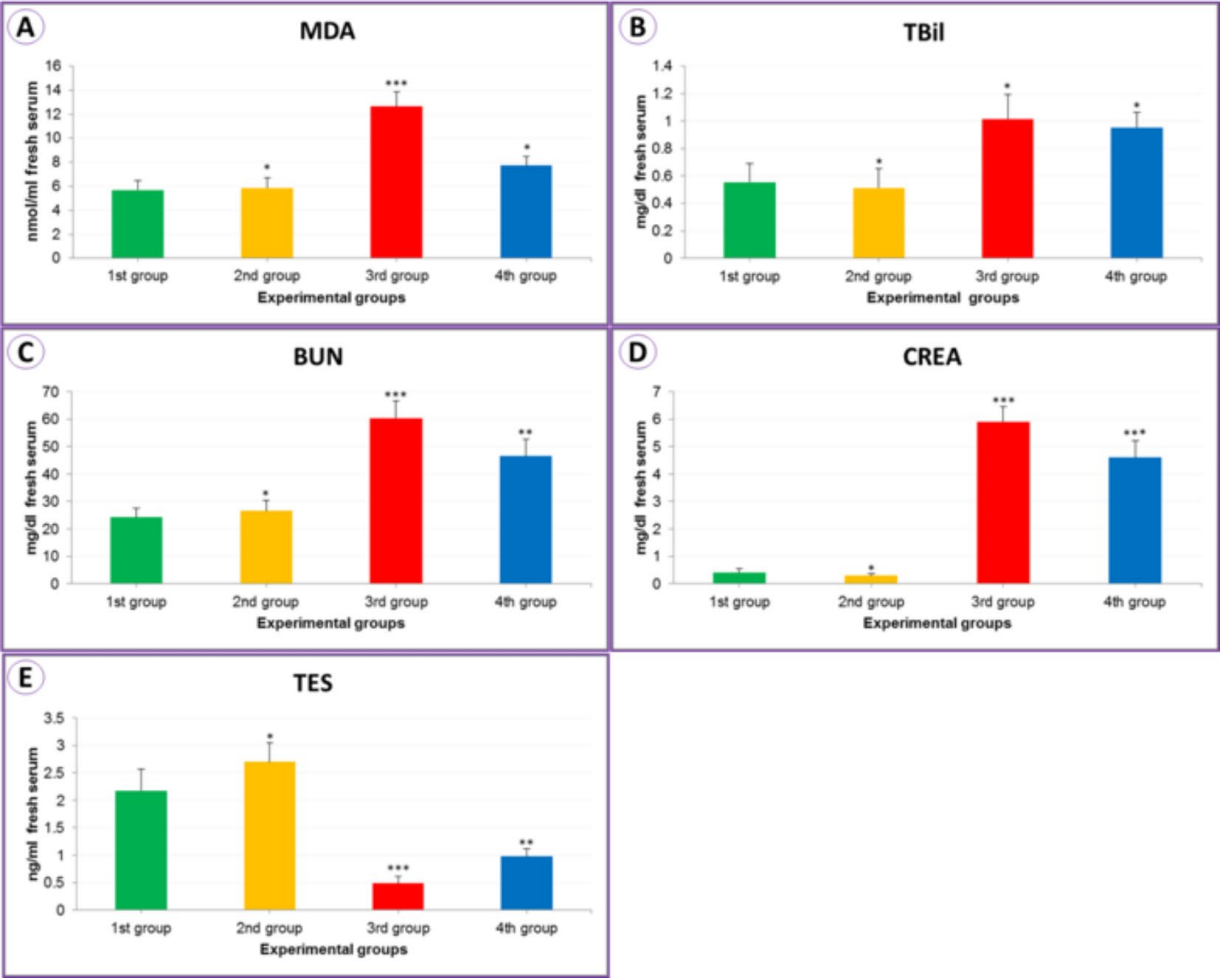
The column bars for MDA (**A**), TBil (**B**), BUN (**C**), CREA (**D**), and TES (**E**) from the mice serum for the experimental current groups.

Malondialdehyde (MDA) (nmol/ml serum) oxidative contents in the serum blood increased significantly (12.63 ± 1.24) (***P < 0.01) in the experimental group exposed to IONPs (3rd group). When treated with seed extract (4th group), there was a significant decrease compared to the control group (1st group) (7.73 ± 0.76), as shown in Table 1; Fig. 3A. As a result of exposure to iron oxide nanoparticles (3rd group), the level of total bilirubin (TBil) (mg/dL of serum) increased to 1.02 ± 0.18 (**P < 0.05) compared to the control (1st group), and this increase decreased as observed as a result of treatment with the seed extract (4th group) (0.95 ± 0.11), as observed in Table 1; Fig. 3B.

In the IONPs group (3rd group), we found a significant increase of 60.39 ± 6.20 (***P < 0.01) in blood urea nitrogen level “BUN” (mg/dL serum) compared to the control experiment 1st group (Table 1 and Fig. 3C), and a level of 5.90 ± 0.56 (***P < 0.01) in creatinine “CREA” levels compared to the control experiment 1st group (Table 1 and Fig. 3D). In the testosterone level “TES” (ng/ml) affected by IONPs (3rd group), its level decreased to 0.49 ± 0.13 (***P < 0.01) when exposed to the PSE with IONPs (4th group); however, there was an improvement and an increase in the level to 0.98 ± 0.14 (**P < 0.05) as illustrated in Table 1; Fig. 3E.

**Table 1:** The malondialdehyde level “MDA” (nmol/ml), total bilirubin level “TBil” (mg/dl), blood urea nitrogen level “BUN” (mg/dl), creatinine level “CREA” (mg/dl), and testosterone level “TES” (ng/ml) from the serum of mice for the experimental current groups.

Experimental groups	Control 1st group	PSE 2nd group	IONPs 3rd group	PSE + IONPs 4th group
MDA	5.67 ± 0.81	5.86 ± 0.83*	12.63 ± 1.24***	7.73 ± 0.76*
TBil	0.55 ± 0.14	0.51 ± 0.14*	1.02 ± 0.18*	0.95 ± 0.11*
BUN	24.19 ± 3.37	26.55 ± 3.87*	60.39 ± 6.20***	46.44 ± 6.21**
CREA	0.41 ± 0.13	0.30 ± 0.06*	5.90 ± 0.56***	4.60 ± 0.62***
TES	2.17 ± 0.40	2.70 ± 0.34*	0.49 ± 0.13***	0.98 ± 0.14**

Mean values ± SE of MDA, TBil, BUN, CREA, and TES level for animals experienced (*n* = 5) for each group.*: Non significantly in comparing to control 1st group (*P* = 0.05), **: Significantly in comparing to control 1st group (*P* = 0.05), ***: High significantly in comparing to control 1st group (*P* = 0.01).

### Histopathological impacts by using the LM

**Fig. 4:**
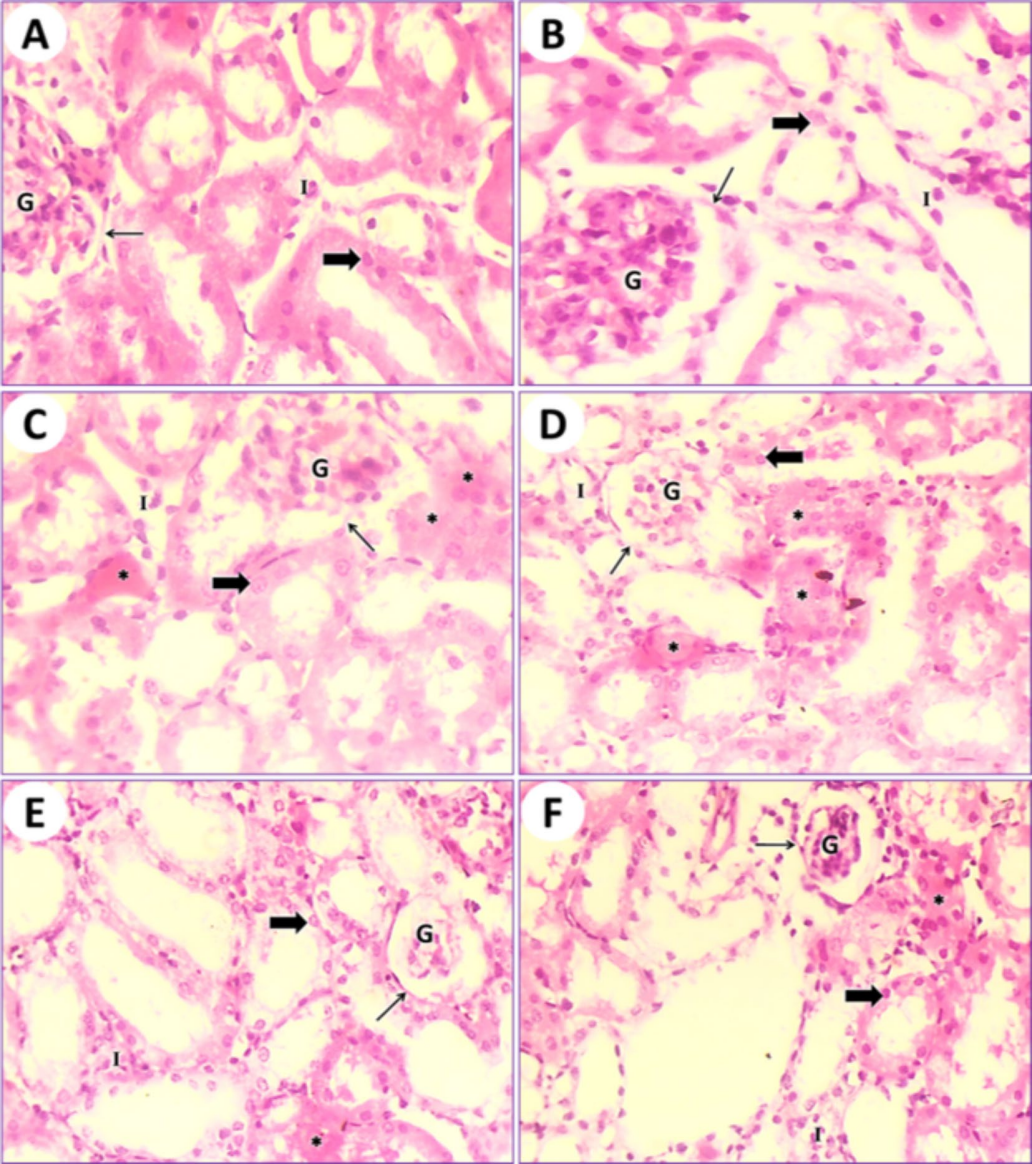
(**A**) Photomicrograph of a transitional section of the kidney from a control mouse (1st group) showing normal Bowman’s capsules (arrow) containing glomeruli and mesangial cells with a normal urinary pole. Normal renal tubules (thick arrow) lined by normal cuboidal epithelium and empty lumen are observed. Also, interstitial cells (I) are normal. (**B**) A transitional section of the kidney from a mouse that was fed a PSE diet (2nd group) displaying normal Bowman’s capsules (arrow) containing glomeruli and mesangial cells (G) with a normal urinary pole. Normal renal tubules (thick arrow) lined by normal cuboidal epithelium and empty lumen are observed. (**C**) A transitional section of the kidney from an IONPs-intoxicated mouse (3rd group) displaying destruction and desquamation of the renal tubular epithelium with the formation of eosinophilic hyaline casts (asterisks) inside the ducts of renal tubules and congestion of interstitial capillaries (I). Bowman’s capsules (arrow) containing glomeruli (G), mesangial cells, and renal tubules (thick arrow) lined with cuboidal epithelium are noted. (**D**) Another part of a transitional section of the kidney from an IONPs-intoxicated mouse (3rd group) shows renal tubules (thick arrow) lined with cuboidal epithelium and mild destruction and desquamation of the renal tubular epithelium with the formation of eosinophilic hyaline casts (asterisks) inside the ducts of renal tubules and congestion of interstitial capillaries (I). Bowman’s capsules (arrow) containing glomeruli (G) and mesangial cells are present. (**E**) A transitional section of the kidney from an IONPs plus PSE-treated mouse (4th group) showing restoration of renal glomeruli (G), mild retrogression of the renal tubular epithelium (thick arrow) with the restoration of normal renal histological architecture, and still some eosinophilic hyaline casts (asterisk) inside the ducts of renal tubules. Indigenous interstitial capillaries (I) are seen. (**F**) Another zone of the previous section displays recovery in renal glomeruli (G), interstitial capillaries (I), recovery of normal kidney histological structure, and still some eosinophilic hyaline casts (asterisk) inside the ducts of renal tubules (thick arrow), [H.&E., 400 X].

In control mice (1st group), the kidney tissue results showed the typical structure of mouse glomeruli, Bowman’s capsules, uriniferous tubules, renal collecting tubules, and cubical cells forming the tubules (Fig. 4A). In mice fed on PSE (2nd group), the kidney tissue showed a structure similar to that of the control (1st group); on the other hand, the renal section of PSE diet mice (2nd group) was close to the renal section of control mice (1st group). Normal Bowman’s capsules contain glomeruli and mesangial cells with normal urinary poles. Normal renal tubules are lined by normal cuboidal epithelium, and the lumen is empty (Fig. 4B). In mice fed on IONPs only (3rd group), the kidney tissue displayed destruction and desquamation of the renal tubular epithelium, with the formation of eosinophilic hyaline casts inside the ducts of the kidney tubules and congestion of interstitial capillaries (Fig. 4C and D). In mice fed on IONPs plus PSE (4th group), the kidney tissue illustrated some restoration of renal glomeruli and mild retrogression of the renal tubular epithelium, with the restoration of normal renal histological architecture (Fig. 4E and F).

**Fig. 5:**
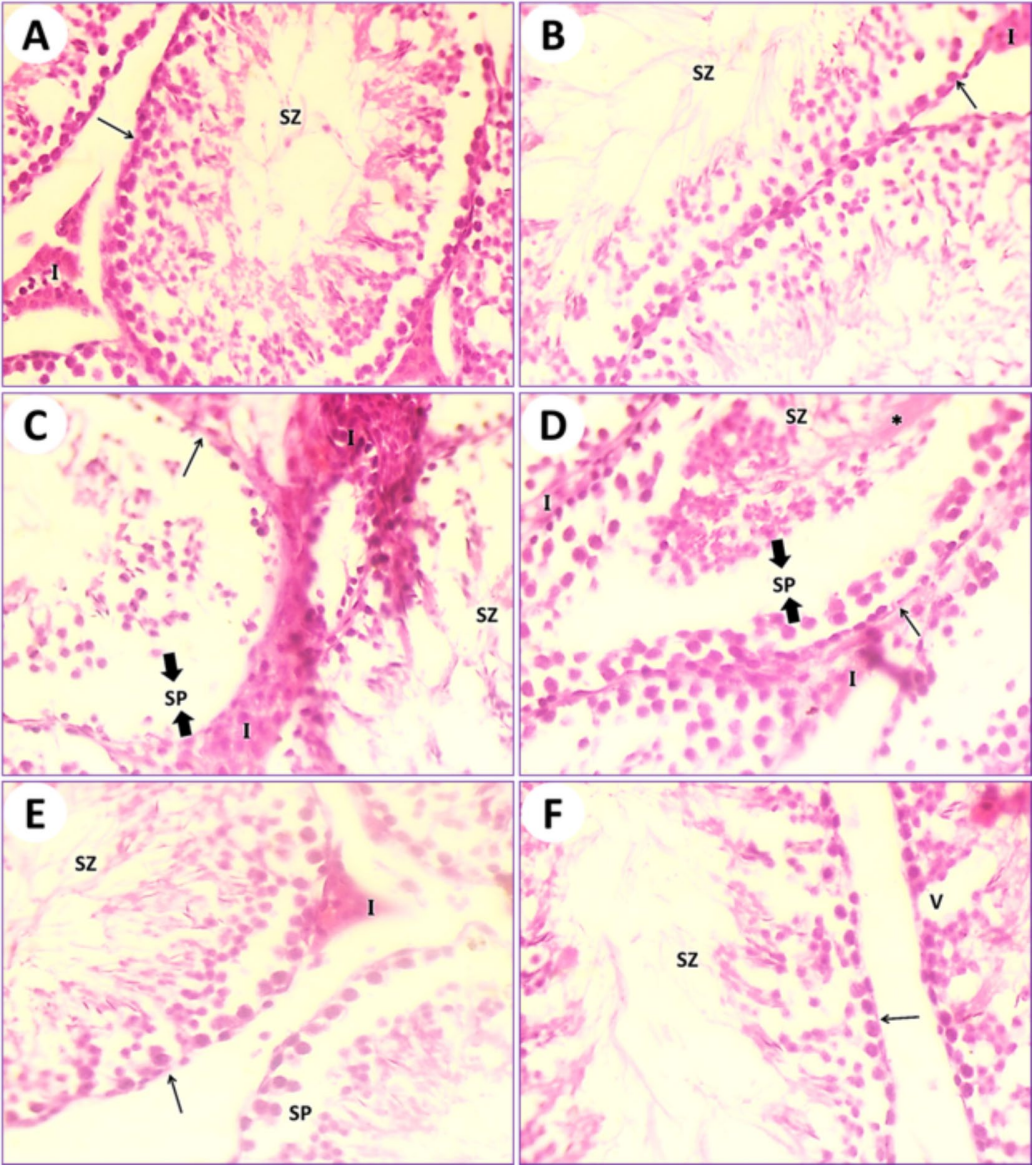
(**A**) Photomicrograph of a transitional section in the testis of an intact control mouse (1st group) showing typical seminiferous tubules (arrow) with normal round densely basophilic spermatogonia and ovoid primary and secondary spermatocytes; also, its lumen is filled with spermatozoa (SZ). Interstitial cells (I) are normal. (**B**) A section of testis from a mouse that was fed a PSE diet (2nd group) displaying the typical manifestation of seminiferous tubules (arrow) with normal round densely basophilic spermatogonia and ovoid primary and secondary spermatocytes, and spermatids; also, its lumen is filled with spermatozoa (SZ). Interstitial cells are normal. (**C**) A section in the testis of an IONPs-exposed mouse (3rd group) displaying severe destruction of seminiferous tubules with necrosis of the lining germinal epithelium (arrow) and complete azoospermia (SZ), with conjugation of interstitial cells (I) due to inflammation occurring in this organ and edema formation in the seminiferous tubules (SP). (**D**) Another field of the previous testicular tissue showing severe disorder of seminiferous tubules with necrosis of the lining germinal epithelium (arrow) and complete azoospermia (SZ), with conjugation of interstitial cells (I) due to inflammation occurring in this organ and edema formation in the seminiferous tubules (SP). (**E**) A section in the testis of an IONPs + PSE treated mouse (4th group) displaying restoration of the normal typical germinal epithelium with mild destruction of spermatogonia (arrow), mild edema, and conjugation of the interstitial cells (I); the duct of seminiferous tubules contains numerous spermatozoa (SZ) and acute inflammation occurs in this organ, along with edema formation in the seminiferous tubules (SP). (**F**) Another section of testis from an IONPs + PSE treated mouse (4th group) detecting recovery of seminiferous tubules (arrow) with normal spermatogonia (SZ) and some vacuoles (V) that have appeared, [H.&E., 400 X].

In control mice (1st group), the testicular section results illustrated the typical architecture of seminiferous tubules and the normal arrangement of spermatogonia and spermatocytes. Many spermatids and spermatozoa were observed. Sertoli cells were few and spaced along the tubule at fairly regular intervals. Thin connective tissues containing interstitial cells appeared in between the seminiferous tubules (Fig. 5A). In mice fed on PSE (2nd group), the testicular section illustrated the testis of control (1st group); on the other hand, the testis section of the PSE diet mouse is similar to the testicular section of the control mouse (1st group). The typical manifestation of seminiferous tubules with normal round densely basophilic spermatogonia and ovoid primary and secondary spermatocytes, as well as spermatids, is present, and its lumen is filled with spermatozoa. Interstitial cells are normal (Fig. 5B). In mice fed on IONPs only (3rd group), the testicular section illustrated severe destruction of seminiferous tubules with necrosis of the lining germinal epithelium, resulting in complete azoospermia. Interstitial cells are expanded due to the destruction of seminiferous tubules and edematous fluid accumulation (Fig. 5C and D). In mice fed on IONPs plus PSE (4th group), the testicular section illustrated some tissue improvement, with mild signs of testis repair, restoration of the normal typical germinal epithelium, mild destruction of spermatogonia, mild edema of the interstitial cells, and ducts of seminiferous tubules containing numerous spermatozoa (Fig. 5E and F).

### Histopathological impacts by using the TEM

**Fig. 6:**
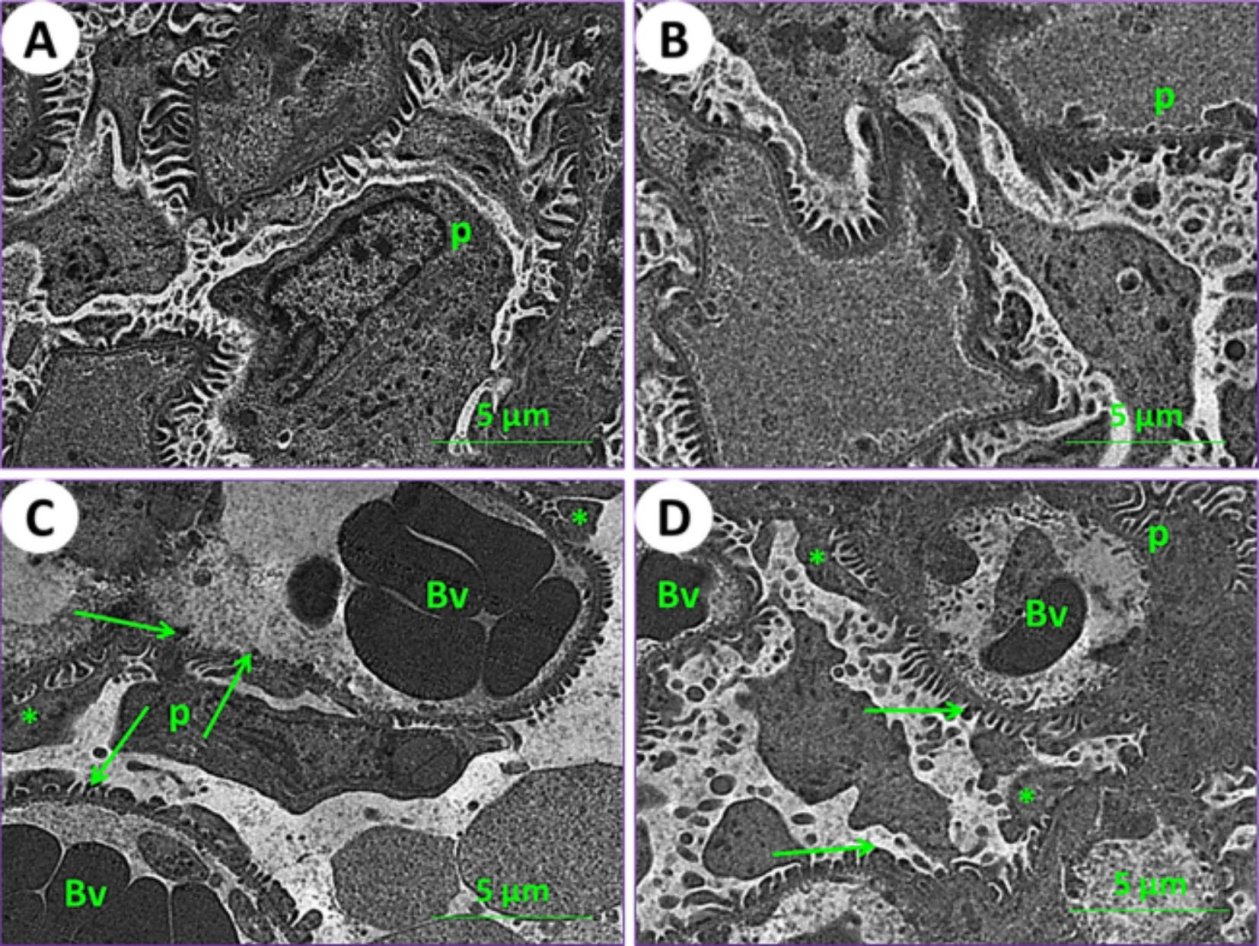
Ultraphotomicrograph plates of the kidney cortex for different experimental current groups: (**A**) Control (1st group), (**B**) PSE (2nd group), (**C**) IONPs (3rd group), and (**D**) PSE + IONPs (4th group). Podocytes (P), glomerular blood vessels (Bv), the processes of podocytes (asterisks), and iron nanoparticles (green arrows) were observed, [TEM, 160000X].

**Fig. 7:**
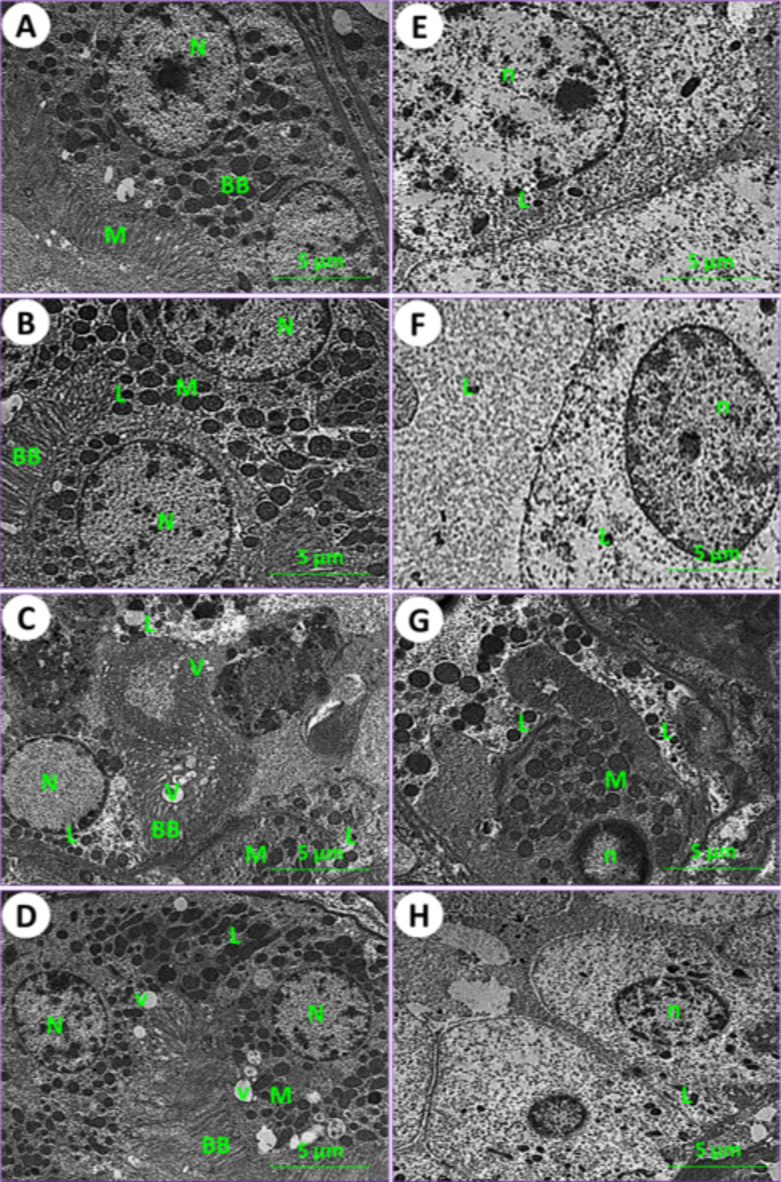
Ultraphotomicrograph plates of the kidney cortex and medulla tubules for different experimental current groups; proximal renal tubules (**A**) Control (1st group), (**B**) PSE (2nd group), (**C**) IONPs (3rd group), and (**D**) PSE + IONPs (4th group). Nucleus (N), lysosomes (L), mitochondria (M), brush border (BB), and vacuoles (V) were discerned. Distal renal tubules (**E**) Control (1st group), (**F**) PSE (2nd group), (**G**) IONPs (3rd group), and (**H**) PSE + IONPs (4th group). Nucleus (n), lymphocytes (L), and mitochondria (M) were elucidated, [TEM, 160000X].

Using an electron microscope (TEM), kidney and testicle ultra-samples were examined for the current experimental groups. In control mice (1st group), the kidney tissue results showed the typical structure of mouse glomeruli, podocytes, glomerulus capsule, proximal and distal tubules (Figs. 6A and 7A, and 7E). In mice fed on PSE, the renal tissues showed similar characteristics to those of the control; on the other hand, the renal section of PSE diet mice (2nd group) was close to the renal section of control mice (1st group), with normal cortex podocytes, glomeruli containing mesangial cells, and a normal renal brush border in proximal and distal tubules (Figs. 6B and 7B, and 7F). In mice fed on IONPs only (3rd group), the renal tissues showed aggregation in the glomerulus, distortion of the podocyte processes, and precipitation of iron nanoparticles (Fig. 6C), along with demolition of the brush border in proximal and distal tubules, numerous and enlarged lysosomes, and emergence of vacuoles (Fig. 7C and G). In mice fed on IONPs plus PSE (4th group), the renal tissues exhibited some improvement in renal glomeruli and mild destruction of the proximal brush border and distal tubules, with restoration of normal renal histological ultra-architectures (Fig. 7D and H).

**Fig. 8:**
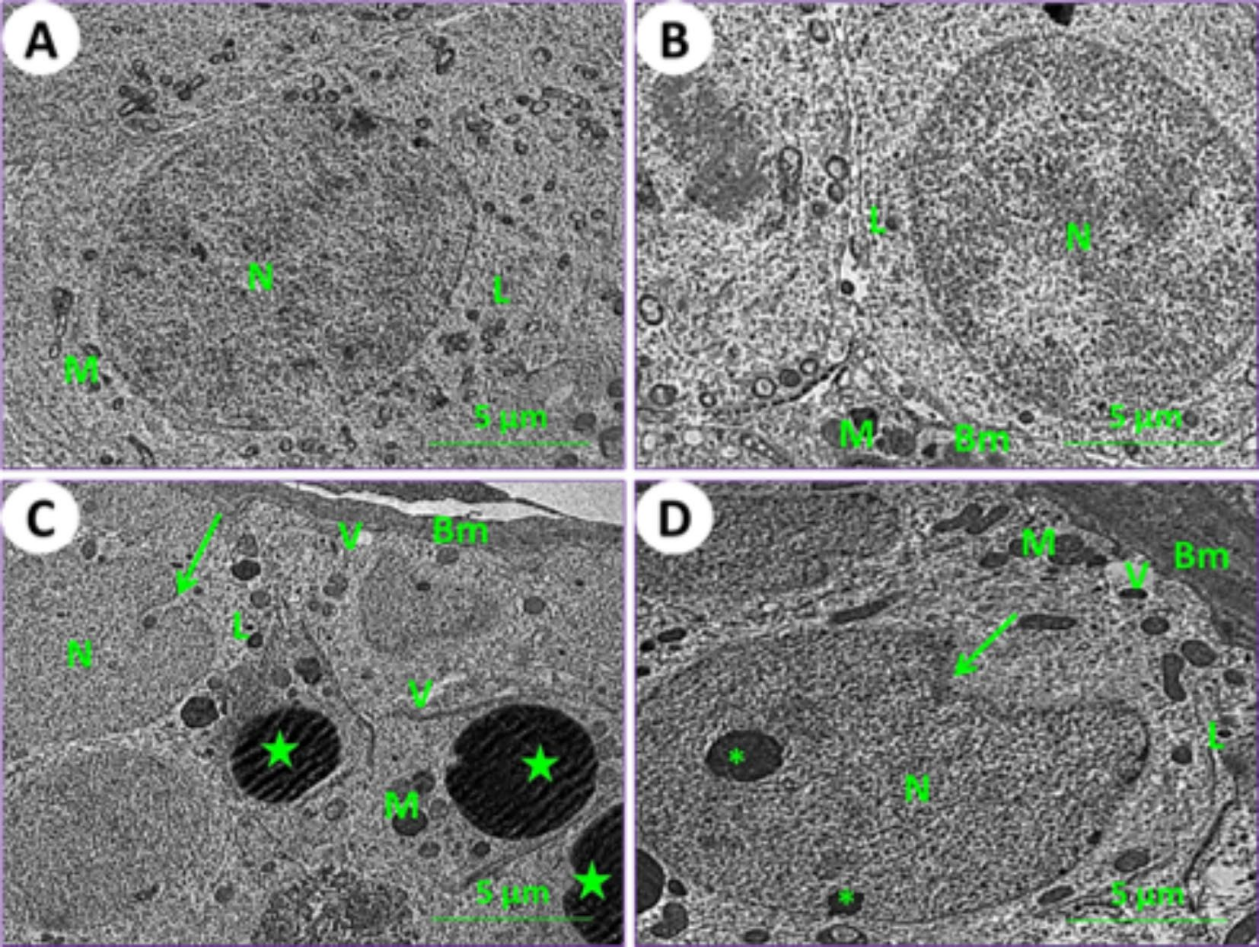
Ultraphotomicrograph plates of the testicular spermatogonia cells for different experimental current groups; (**A**) Control (1st group), (**B**) PSE (2nd group), (**C**) IONPs (3rd group), and (**D**) PSE + IONPs (4th group). Nucleus (N), lysosomes (L), mitochondria (M), basement membrane (BM), vacuoles (V), distortion of chromatin (Asterisks), and lipid spherical (Green stars) were shown, [TEM, 160000X].

In control mice (1st group), the testicular section results showed the typical architecture of seminiferous tubules, and a normal arrangement of spermatogonia cells was noticed (Fig. 8A). In mice fed on PSE (2nd group), the testicular section showed a similar appearance to that of the control; on the other hand, the testicular section of the PSE diet mouse was similar to that of the control mouse, with normal manifestation of spermatogonia cells (Fig. 8B). In mice fed on IONPs only (3rd group), the testicular section showed severe destruction of spermatogonia cells, thickening of the basement membrane, appearance of vacuoles, numerous enlarged lysosomes, distortion of chromatin, and lipid spherules (Fig. 8C). In mice fed on IONPs plus PSE (4th group), the testicular section illustrated some tissue improvement, with mild signs of testicular repair and mild destruction of spermatogonia cells; some vacuoles were found (Fig. 8D).

### Histochemical impacts by using the LM

**Fig. 9:**
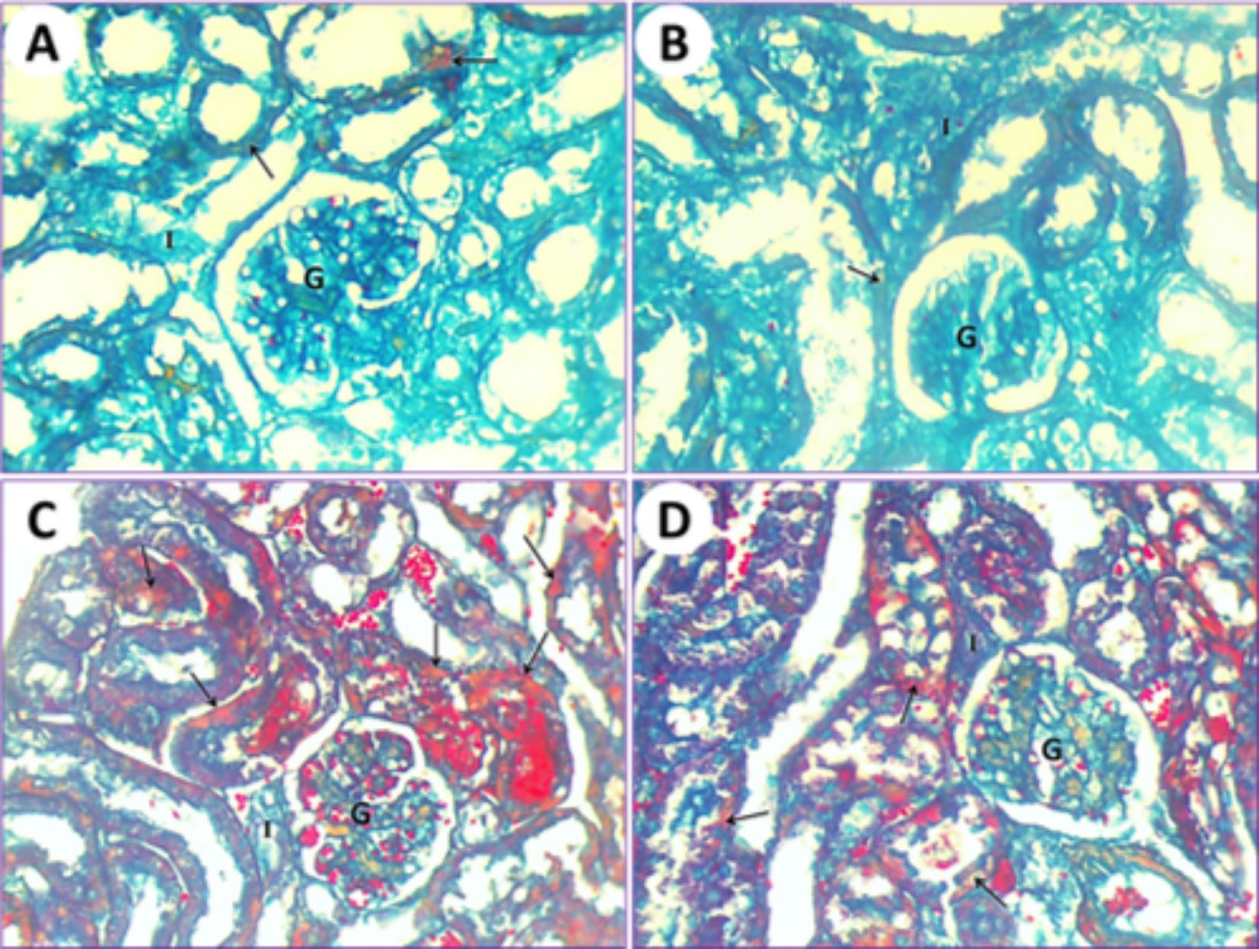
(**A**) Kidney section by light microscope from normal healthy albino mice (1st group) represents the natural distribution of collagen fibers (arrow) in the cytoplasm of the epithelium. Glomerulus (G) and interstitial cells (I) are visible. (**B**) Kidney section by light microscope from PSE-treated (2nd group) mice shows a normal distribution of collagen fiber contents that is similar to the control. Glomerulus (G) and interstitial cells (I) are noted. (**C**) Kidney section by light microscope from IONPs-induced mice (3rd group) detects a marked increase in collagen fiber content inside the epithelium and lumen of renal tubules (arrows), glomerulus (G), and interstitial cells (I). (**D**) Kidney section by light microscope from IONPs plus PSE-treated mice (4th group) displays a more diminished Mallory trichrome reaction for collagen fibers (arrows) around the glomerulus (G) and interstitial cells (I), [MTS, 400 X].

The application of the Mallory trichrome reaction on normal mice (1st group) revealed the natural distribution of collagen fibers in the renal tissue (Fig. 9A). Kidneys of mice treated with PSE (2nd group) showed normal or slight amounts of collagen fibers that are similar to those in the first normal group (Fig. 9B). Renal tissue from mice subjected to IONPs exposure (3rd group) showed a gradual increase in collagen fiber content as observed with Mallory trichrome staining (Fig. 9C). After treatments with IONPs plus PSE (4th group), the collagen fiber content in the kidney was restored to a lower level but still less than that of the control (Fig. 9D).

**Fig. 10:**
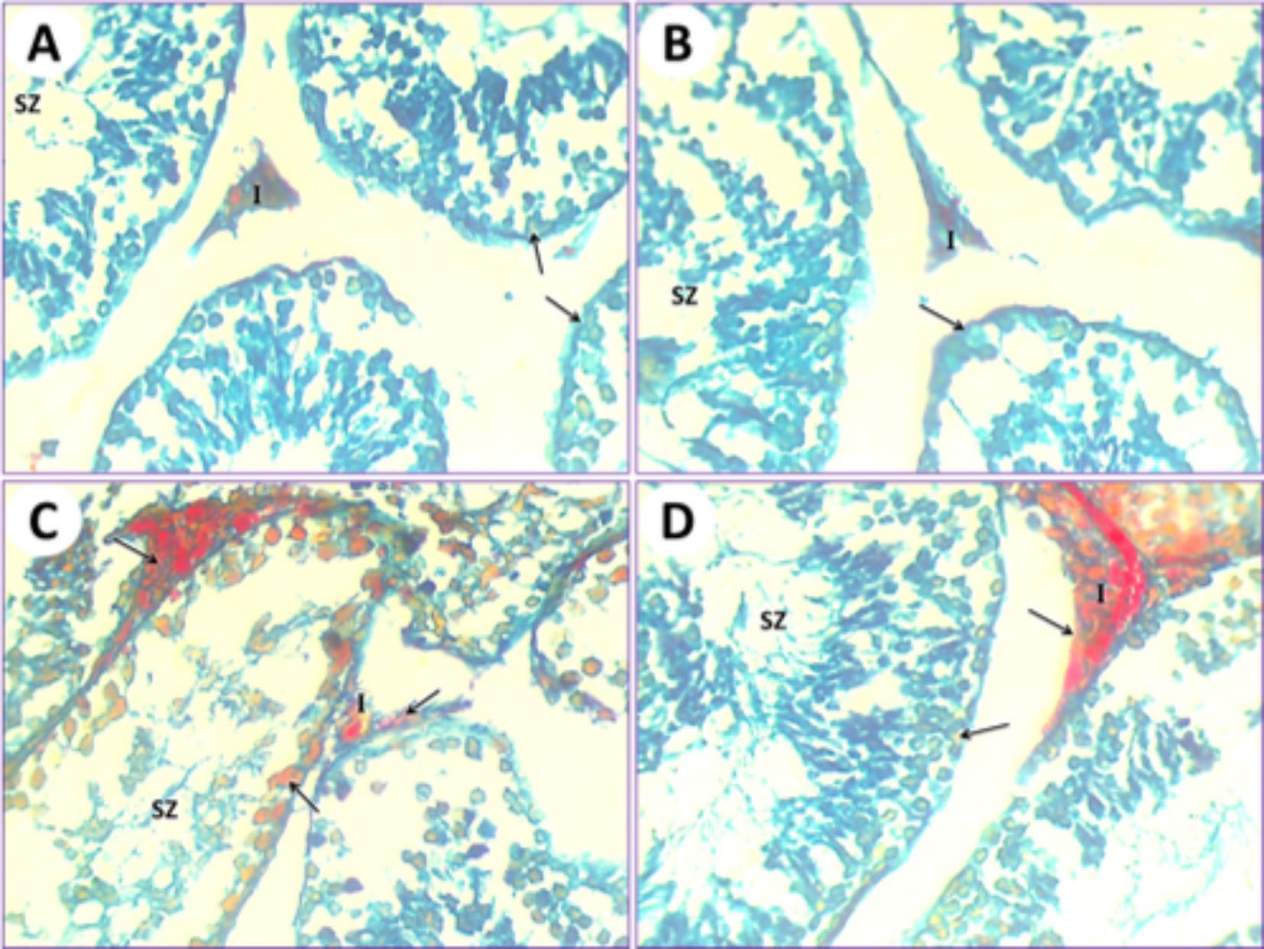
(**A**) The testis of the control mouse (1st group) shows normal collagen fiber distribution in seminiferous tubules (arrows); its lumen is plugged with spermatozoa (SZ) and interstitial cells (I). (**B**) The testis from the mouse fed a PSE diet (2nd group) displays normal collagen fiber content (arrow) in the typical seminiferous tubules with different spermatogonia and spermatocytes, and its lumen is plugged with spermatozoa (SZ) and interstitial cells (I). (**C**) The testis of the IONPs-exposed mouse (3rd group) displayed an increased Mallory trichrome reaction for collagen fibers (arrows), expansion of interstitial cells (I), and congestion, along with severe destruction of seminiferous tubules and necrosis of the lining germinal epithelium, resulting in azoospermia (SZ) in some seminiferous tubules. (**D**) The testis of the IONPs plus PSE treated mouse (4th group) displays some restoration of collagen fibers (arrow) at the location of the germinal epithelium of seminiferous tubules, with the lumen filled with spermatozoa (SZ) and interstitial cells (I), [MTS, 400 X].

In control mice (1st group), the testicular section results illustrated that the seminiferous tubules were typical, and their ducts were plugged with spermatozoa and interstitial cells. The collagen fibers are markedly stained by Mallory trichrome (Fig. 10A). In mice fed on PSE extract (2nd group), the testicular section illustrated the testis of the control (1st group); on the other hand, the testis section of the PSE diet mouse (2nd group) was similar to the testis section of the control mouse (1st group). The typical manifestation of seminiferous tubules shows natural contents of collagen fibers in the interstitial cells, typical seminiferous tubules with different spermatogonia and spermatocytes, and their lumen is plugged with spermatozoa (Fig. 10B). In mice fed on IONPs only (3rd group), the testicular section displayed a severe increase in collagen fiber content in the seminiferous tubules, with necrosis of the lining germinal epithelium and congestion appearing in interstitial cells (Fig. 10C). In mice fed on IONPs plus PSE (4th group), the testicular section illustrated some tissue improvement, with some restoration of collagen fibers in the interstitial cells, and normal germinal epithelium of seminiferous tubules with the lumen filled with spermatozoa (Fig. 10D).

## Discussion

There is a need for concern and a strike as a result of man being subjected to the danger of iron nanoparticles in the medical, industrial, or environmental fields, as they cause synergistic toxicities^6,7,28^. The current experimental study has proven what precedent studies have shown: the toxicity of being subjected to iron nanoparticles causes systemic responses in the tissues of mice. They also suffer from indicators of damage and increased formation of free radicals (ROS), which is due to the weakening of the antioxidant and metabolic mechanisms^5,7,8,16,17^.

The current experimental study clarified the effective improvement of PSE on the toxicity of iron nanoparticles in the kidney and measles tissues of laboratory mice. The results demonstrated an imbalance in blood biomarker chemistry levels, including malondialdehyde (MDA), total bilirubin (TBil), blood urea nitrogen (BUN), creatinine (CREA), and testosterone, as well as histological and histochemical examinations (LM and TEM) of kidney and testicular tissues in mice. INOPs (3rd group) caused damaging effects on blood biomarkers and experimental tissues, increasing inflammation and affecting metabolic changes.

Extract of pomegranate can modify, correct, and improve textural defects caused by Fe_2_O_3_ nanoparticles. The extract components of pomegranate have been shown to affect cell proliferation, and the inflammation effects are reduced^29^. Previous research has supported the beneficial impacts of pomegranate extract on hepatic, lung, renal, and testicular cells^8,23,30–32^. The antioxidant impacts of pomegranate are a result of its content of ascorbic acid, polyphenols, carotene, and vitamin E, providing a broad protective spectrum of effects against many free radicals caused by iron nanoparticles.

Clear new research is very important and required because it can pave the way and further explore new studies in the fields of human health and occupational settings. Extrapolation of study results is important for subsequent application to humans; therefore, there may be subsequent careful studies of factors such as pharmacokinetics, translation of appropriate doses depending on the case of risk, and differences in drug metabolism and response.

## Conclusion

The positive extract effects of *Punica granatum* seed (PSE) on kidney and testicular toxicity in mice influenced by nanoparticles of iron oxide (INOPs) represent an important and promising research avenue. Using a comprehensive approach that includes in vivo toxicity evaluation and analyses, the potential of PSE to reduce the harmful effects of INOPs on vital organs can be explored systematically. Ultimately, such evaluations could contribute to the development of novel strategies for the protection and mitigation of toxicity related to nanoparticles and enhance our understanding of the important role of natural compounds in reducing industrial and ecological health risks.

## Data Availability

All data regarding this study are presented in the paper.
